# An Effective Palmprint Recognition Approach for Visible and Multispectral Sensor Images

**DOI:** 10.3390/s18051575

**Published:** 2018-05-15

**Authors:** Abdu Gumaei, Rachid Sammouda, Abdul Malik Al-Salman, Ahmed Alsanad

**Affiliations:** 1 Department of Computer Science, College of Computer and Information Sciences, King Saud University, Riyadh 11543, Saudi Arabia; rsammouda@ksu.edu.sa (R.S.); salman@ksu.edu.sa (A.M.A.-S.); 2 Department of Information Systems, College of Computer and Information Sciences, King Saud University, Riyadh 11543, Saudi Arabia; aasanad@ksu.edu.sa

**Keywords:** security, visible and multispectral palmprint images, HOG-SGF feature extraction, auto-encoder, regularized extreme learning machine

## Abstract

Among several palmprint feature extraction methods the HOG-based method is attractive and performs well against changes in illumination and shadowing of palmprint images. However, it still lacks the robustness to extract the palmprint features at different rotation angles. To solve this problem, this paper presents a hybrid feature extraction method, named HOG-SGF that combines the histogram of oriented gradients (HOG) with a steerable Gaussian filter (SGF) to develop an effective palmprint recognition approach. The approach starts by processing all palmprint images by David Zhang’s method to segment only the region of interests. Next, we extracted palmprint features based on the hybrid HOG-SGF feature extraction method. Then, an optimized auto-encoder (AE) was utilized to reduce the dimensionality of the extracted features. Finally, a fast and robust regularized extreme learning machine (RELM) was applied for the classification task. In the evaluation phase of the proposed approach, a number of experiments were conducted on three publicly available palmprint databases, namely MS-PolyU of multispectral palmprint images and CASIA and Tongji of contactless palmprint images. Experimentally, the results reveal that the proposed approach outperforms the existing state-of-the-art approaches even when a small number of training samples are used.

## 1. Introduction

Biometric technology is a valuable tool that is used for security purposes in many applications [1]. In recent years, it has gained more attention worldwide. Several biometric traits, including face, gait, iris, key-stroke, fingerprint, and palmprint have been widely studied and developed depending on the application domain most suitable for them [2,3,4]. Compared to other biometrics, palmprint has a low distortion, strong stability, and high uniqueness [5]. Previously, palmprint images were only acquired in grayscale formats by using traditional natural light imaging systems. Recently, a new technique, called multispectral palmprint imaging, is used to increase the performance and accuracy of traditional natural light imaging systems. Systematically, the multispectral palmprint technique is able to capture the palm in a variety of spectrums, generally from 3 to 10, and typically 4 spectrums which are red, green, blue, and near-infrared (NIR) spectral bands. Therefore, in comparison with the natural light image, each spectral band highlights different features, making it possible to obtain more information for improving a palmprint recognition system [6].

However, palmprint patterns in both natural light and multispectral imaging systems may be affected by several factors, such as changes in orientation, variations in illumination, and the sensors’ noise, which may lead to misclassification. Variations in illumination and orientation of multispectral palmprint images can greatly affect the capability of systems to recognize individuals. Different approaches have been developed to overcome these challenging issues by proposing various feature extraction, reduction, and matching methods. These approaches can be categorized into four classes: line-based, statistical-based, subspace-based, and coding-based approaches.

Line-based approaches are used to extract palm lines by developing edge detectors, or by using existing edge detection methods. Some researchers, such as Han et al. [7], have proposed a method for extracting line features of palm images based on morphological operations with a Sobel edge detector. Wu et al. [8] applied the Sobel mask to generate histograms by computing the magnitudes of palm lines and projecting these magnitudes along both the x and y axes.

Although the line-based approaches have proven to attain a satisfactory performance, these approaches require a very high-resolution image of the palmprint. To acquire such a resolution is unrealistic and requires excessive cost sensors and devices.

Statistical-based approaches have also been explored in several studies of palmprint recognition and achieved desirable results [9,10,11]. There are many statistics used in these approaches, such as Zernike moments, Hu moments, energy, mean, variance, standard deviation, and histograms of local binary patterns. Several transforms have also been used to extract better unique features. A previous method [12] applied the wavelet transformation to transform a palm image into the wavelet domain, and then computed the mean and variance of each block to generate a feature vector. Jing at al. [13] proposed a method known as the two-phase test sample representation (TPTSR), and used it for palmprint recognition. Yong et al. [14] proposed a method to adjust the coarse to fine k-nearest neighbor classifier (CFKNNC) for improving the recognition accuracy. The limitation of this study is that the CFKNNC has more steps and appears to be more complicated than the CKNNC. A nearest feature space (NFS) classifier is also used to compute the distance between the feature space of the training set of each class and the testing set. A novel palmprint recognition method proposed by Zhang et al. [15] uses the linear combination of all training sets in the feature space to represent the test set based on RBF kernel function mapping. Researchers in the area of palmprint recognition have also applied a variety of different texture feature methods, such as Gaussian derivative filters, Gabor wavelet, Fourier transform, and the wavelet transform. Xingpeng et al. [16] proposed a novel method using a quaternion matrix for palmprint recognition. A discrete wavelet transform (DWT) and principal components analysis (PCA) are utilized to extract textural features from the quaternion matrix. Following that, the sum of two distances between features is used for recognition decision. However, the use of the statistical-based approaches is very sensitive to changes in illuminations and not robust against the sensors’ noise.

In the literature of palmprint recognition, subspace-based approaches have been developed to make the classification task easier by finding the discriminative subspace of features. There are several representative subspace learning approaches such as linear discriminant analysis (LDA), PCA, and independent component analysis (ICA). Recently, novel and robust representative subspace methods, such as robust discriminant regression (RDR) [17] and low-rank representation (LRR) [18], are proposed for effective feature extraction. On the other hand, Wen et al. [19] proposed a novel framework for classification based on the unsupervised optimal feature selection (UOFS) model. The authors introduced into the model a projection matrix with L2, 1-norm regularization for robust subspace learning and selection. Fei et al. [2] proposed a method, named (LRRIPLD), to integrate the LRR with adaptive principal line distance of palmprint for identification from contactless palm images. The first use of PCA in palmprint recognition was by Lu et al. [20], the original palmprint images are transformed into a small feature space set, called an eigenpalm. Actually, an eigenpalm represents the PCA eigenvectors of the palmprint images in the training dataset. In this method, Euclidean distance is used for matching. Similarly, some studies have applied both vertical and horizontal two-dimensional LDA (2DLDA) for extracting the Gabor features. After that, a distance-based adaptive strategy was used to combine the vertical and horizontal features [21].

In [22], Xu et al. introduced a novel approach for multispectral palmprint recognition using a multiclass projection extreme learning machine (MPELM) with digital a Shearlet transform. In this approach, a singular value decomposition (SVD) is used to compute a projection matrix from the training dataset, and then the *k*th singular vector is selected corresponding to the largest values of a singular matrix. Lu et al. [23] presented a multispectral palmprint recognition approach using a fast and adaptive bidimensional empirical mode decomposition (FABEMD) method with a tensor flow extreme learning machine (TFELM) classifier. In this approach, the multispectral images are decomposed into their bidimensional intrinsic mode functions by the FABEMD method; then the fusion coefficients are constructed at the decomposition level using the weighted Fisher criterion method. Experimental results [22,23] have demonstrated the capability of ELM classifier to recognize the palmprint patterns. However, ELM is not robust to translation, rotation, and other changes of palmprint templates and needs some regularization parameters for generality during the training phase. The authors of another paper [24] proposed a novel method that combines two algorithms of feature extraction; spectral regression kernel discriminant analysis (SR-KDA) was utilized for dimensionality reduction and k-nearest neighbor (KNN) classifier was employed for classification. Rida et al. [25] proposed a palmprint identification method based on the ensemble of sparse representations classifier (SRC); however, SRC is very slow and is still affected by changes in palmprints in the case of small number of training data samples. In general, although the subspace-based approaches perform well for palmprint recognition, they still lack the robustness against variations in palmprint templates, especially when they use weak classifiers for matching and recognition.

Coding-based approaches have been widely used in the field of palmprint recognition. There are several methods for features coding that have been described in the literature, such as palm code [5], fusion code [26], ordinal code [27], competitive code [28], log-Gabor code [29], and collaborative representation competitive code (CR_CompCode) [30]. For example, the fusion coding method encodes the phase of six Gabor filter responses and uses them for competition. Finally, the orientation of the maximum response is selected as a feature. Jia et al. [31] proposed a method called the robust line orientation code (RLOC). This method extracts the palmprint orientations using a modified finite Radon transform. The extracted orientations feature vector is employed as a competitive code. The new set of features is generated using the mixture of Gabor filter and LBP-HF descriptors. Guo et al. [32] proposed a new approach, named the Binary Orientation Co-occurrence Vector (BOCV) for extracting more orientation features. The approach convolves the six Gabor filters with the palmprint image and encodes the results into six orientation features. To further improve the performance of BOCV, Zhang et al. [33] proposed an Extend BOCV (EBOCV) approach which removes the fragile bits from the resulted binary vector. Fei et al. [34] proposed a palmprint recognition approach using a half-orientation code (HOC), in which the extraction of half-orientation defines a bank of half-Gabor filters. Another approach [35] proposed a double-orientation code (DOC) using Gabor filters and the nonlinear matching method. Other proposed approaches [34,35] have been evaluated by the MS-PolyU database of multispectral palmprints. Hong et al. [6] proposed a HOG-based feature extraction method, named the Block-wise histogram of orientated gradient (BHOG). The authors fused the BHOG features to improve the performance of palmprint recognition. However, the BHOG method ignores the global effect of changes in illumination and shadowing between blocks of the extracted feature vector. More work has been introduced to improve the palmprint recognition system; Hao et al. [36] proposed a method for the identification of individuals using the orthogonal line ordinal features (OLOF) of palmprint images. Morales et al. [37] fused the extracted points of the scale-invariant feature transform (SIFT) method with the orientation features of the contactless palmprint images, and used them for matching. Doublet et al. [38] implemented a method for contactless palmprint recognition, named (OLOF_SIFT). In the OLOF_SIFT method, the extracted points of OLOF and SIFT from palm images are fused to perform the palmprint recognition. Michael et al. [39] introduced a feature extraction approach based on the local binary pattern (LBP) method for palmprint recognition.

Even though all previous approaches and methods improved the performance of palmprint recognition by extracting distinctive features, such as OLOF, SIFT, and LBP, the improvements are not significant and are relatively limited compared to the processing time and size of extracted features. Moreover, the enormous size of the features makes the palmprint recognition model suffer from the overfitting problem which leads to misrecognition. Therefore, extracting more distinguishable and distinct characteristics of palmprints should be considered in the feature extraction step.

In this paper, an effective palmprint recognition approach is developed with the following contributions. First, a new hybrid feature extraction method, named HOG-SGF, is proposed to extract the palmprint features efficiently and effectively. The HOG-SGF method is able to combine the HOG features with the standard deviation and mean features of SGF responses, summarizing the palmprint features at different rotation angles. Second, an optimized auto-encoder (AE) is specially used for the dimensionality reduction of palmprint features. AE works better than PCA and LDA methods for modeling the distribution of multimodal characteristics and nonlinearity of features. Third, a fast and robust regularized extreme learning machine (RELM) classifier is applied for palmprint recognition. In the RELM classifier, a Frobenius norm is exploited as a regularization parameter in a trade-off between the approximated error and the regularized degree of the training samples to improve the generalization of changes in palmprint images. Finally, a large set of experiments was conducted on three palmprint databases collected from touch-free and touch-based acquisition devices in visible and multispectral imaging systems to evaluate the effectiveness, robustness, and efficiency of the proposed approach. Moreover, the experimental analysis of multispectral palmprint images was performed to show the advantages of band combination for improving the accuracy of palmprint recognition.

The rest of the paper is structured as follows. Section 2 gives a brief review of the AE method. Section 3 describes the RELM algorithm. Section 4 presents the proposed palmprint recognition approach based on AE and RELM with an efficient HOG-SGF descriptor. A comprehensive set of experiments on three benchmark databases of palmprint is demonstrated in Section 5. Finally, concluding remarks and future work are summarized in Section 6.

## 2. Auto-Encoder (AE)

**Figure 1:**
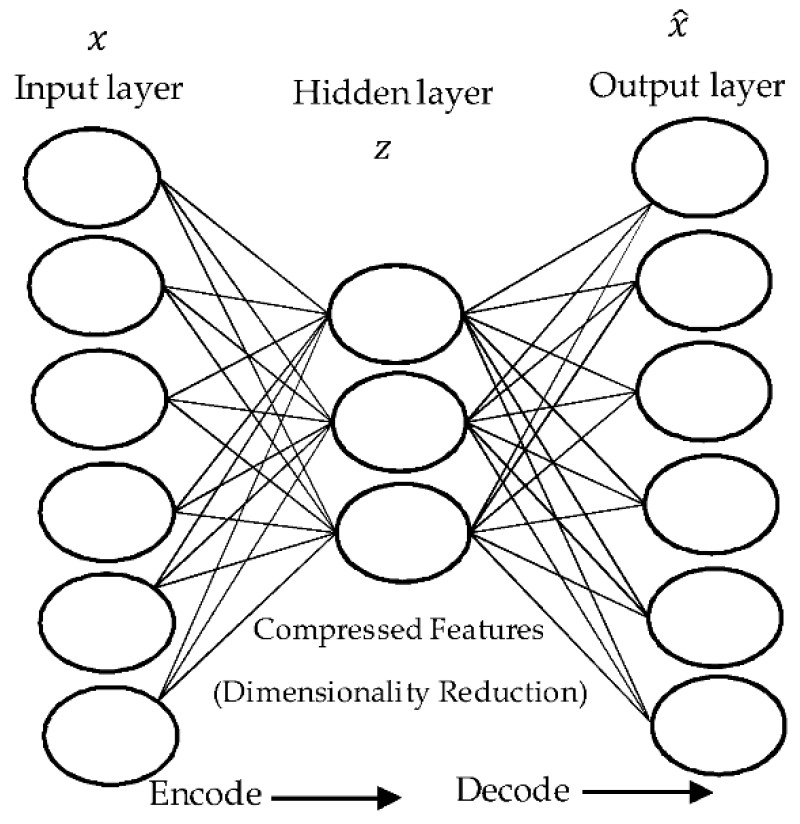
An autoencoder (AE) with a single hidden layer.

Auto-encoder (AE) is a feedforward neural network (FNN) which has the same number of neurons in the input and output layers. It is used for the purpose of unsupervised learning as an effective encoding method [40,41]. To reproduce the outputs from the inputs, it encodes the inputs into undercomplete or overcomplete representations then tries to reconstruct the outputs from the representations of inputs. These representations are often computed using nonlinear or linear functions. In undercomplete representation, the number of neurons in the hidden layer can be less than the input which means that the AE is used to learn more representative features for dimensionality reduction as used in our work. While, overcomplete representation is used to transform the input data to another feature space that could be more discriminative. A typical form of AE with a single hidden layer is shown in Figure 1.

As we mentioned before, the number of nodes in the input and output layers is the same, making it possible for the autoencoder to reconstruct its own inputs instead of predicting a target value from a given input value. Accordingly, the AE is a kind of unsupervised learning. In this case, one can evaluate the reconstruction error between input nodes and output nodes using a specific loss function. More precisely, AE always consists of two parts, the encoder and decoder. The encoder part can map the input vector, $X\epsilon {ℛ}^{N},$ to its hidden (or latent) representation, $Z\epsilon {ℛ}^{K},$ as:

(1) $$Z=\phi_{1}\left(A_{1}X+b_{1}\right),$$

where $\phi_{1}\left(\cdot \right)$ is an activation function, $A_{1}$ is an *N* × *K* matrix of weights, *N* is a number of input nodes, *K* is a number of hidden nodes, and $b_{1}$ is a bias vector. As an alternative, the decoder can map the hidden representation to the output reconstruction as:

(2) $$\hat{X}=\phi_{2}\left(A_{2}X+b_{2}\right),$$

where $\phi_{2}\left(\cdot \right),$ $A_{2},$ and $b_{1}$ are the activation function, the matrix of weights and the bias vector, respectively. The set of these parameters, $\theta =\left\{A_{1},\;b_{1},A_{2},\;b_{2}\right\}$ can be learned by minimizing the average reconstruction error between input and output over a suitable training set. Usually, the average of Euclidean distance is applied as loss function:

(3) $$L\left(\theta \right)={\left|X-\hat{X};\theta \right|}^{2}$$

AE has a bottleneck structure when the number of hidden nodes is less than the number of inputs (*K* < *N*), and the optimization process can be carried out using stochastic gradient descent. In this case, it represents the input with a smaller number of hidden nodes to preserve the information content as much as possible. If a linear activation function is used, AE works as a PCA and provides a linear representation of data. On the other hand, nonlinear activation functions, such as hyperbolic tangent, rectified linear unit (ReLU), or a sigmoid function can be used to make the AE works better than PCA and LDA methods for dealing with multimodal characteristics of the input distribution [42]. Other advantages of AE can be summarized as follows:

▪ AE, which is a type of neural network, can be easily used in a parallel fashion.
▪ The pretrained AE model with its initial weights can be utilized to produce more robust latent representations of the input data.
▪ The nature of AE’s learning algorithms, such as online or iterative gradient descent, can allow us to train the AE model by batches compared to other dimensionality reduction methods which require the whole data in the training phase.

## 3. Extreme Learning Machine (ELM)

An extreme learning machine (ELM) is a feedforward neural network with a single hidden layer. The input weights of ELM are chosen randomly and the output weights are computed mathematically. ELM is adopted in our work because it is fast for training and testing which makes it more suitable for a real time palmprint recognition system. For any *N* distinct samples $\left(x_{i},t_{i}\right)\epsilon {ℛ}^{N\times K}$, the ELM with *K* hidden nodes and an activation function, *g*(*x*), are mathematically modeled as:

(4) $$o_{j}=\overset{K}{\underset{i=1}{\sum }}\beta_{i}g\left(w_{i}\cdot x_{j}+b_{i}\right),\;j=1,\ldots ,N,$$

where $w_{i}={\left[w_{i1},\;w_{i2},\ldots ,w_{iK}\right]}^{T}$ is the weight vector which connects each hidden node *i* with all input nodes, $w_{i}\cdot x_{j}$ represents the inner product of $w_{i}$ and $x_{j}$, $b_{i}$ is the threshold of the *i*th hidden nodes, and $\beta_{i}={\left[\beta_{i1},\;\beta_{i2},\ldots ,\beta_{iN}\right]}^{T}$ is the weight vector between the *i*th hidden nodes and the output nodes. These *N* samples can be approximated with zero error by:

(5) $$\sum_{j=1}^{N}\|o_{j}-t_{j}\|=0,$$

where $t_{j}$ can be computed using the following equation:

(6) $$t_{j}=\overset{K}{\underset{i=1}{\sum }}\beta_{i}g\left(w_{i}\cdot x_{j}+b_{i}\right),\;j=1,\ldots ,N,$$

The above equations can be reformulated shortly as:

(7) Hβ=T,

where

(8) $$H\;={\left[\begin{array}{ccc} g\left(w_{1}\cdot x_{1}+b_{1}\right) & \cdots & g\left(w_{K}\cdot x_{1}+b_{K}\right)\\ \vdots & \cdots & \vdots \\ g\left(w_{1}\cdot x_{N}+b_{1}\right) & \cdots & g\left(w_{K}\cdot x_{N}+b_{K}\right) \end{array}\right]}_{N\times K}$$

(9) $$\beta ={\left[\begin{array}{c} \beta_{1}^{T}\\ \vdots \\ \beta_{K}^{T} \end{array}\right]}_{K\times N}\;\mathrm{and}\;T={\left[\begin{array}{c} t_{1}^{T}\\ \vdots \\ t_{N}^{T} \end{array}\right]}_{N\times K}$$

*H* is the output matrix of the hidden layer where each i column of H represents the output of the i. hidden node related to the inputs, $x_{1},\;x_{2},\ldots ,x_{N}.$ The parameters of hidden nodes with nonzero activation functions can be fixed randomly, and the output weights can be computed on any input data sets [43,44]. So, Equation (7) becomes a linear system where the output weights, β is estimated as:

(10) $$\hat{\beta }=H^{+}T,$$

where $H^{+}$ represents the pseudoinverse of the output matrix of the hidden layer, H. Thus, the output weight vector, β, is computed in a single step to avoid the long training procedure. In this case, the parameters of the network are tuned in iterative manner using the selected parameters.

We can say that the ELM is equal to SVM from the perspective of optimization methods, but ELM has fewer restrictions of optimization and higher speed for training and testing in the case of multiclass classification with high dimensionality of features [45].

### Regularized Extreme Learning Machine (RELM)

Assume that $X\epsilon {ℛ}^{N\times K}$ is a matrix of training examples. The ELM with *K* hidden nodes and activation function, *g(x)* are mathematically modeled by Equation (4).

The solution of the ELM linear system is equivalent to finding the solution of least-squares when the estimated weight, $\hat{\beta },$ satisfies the following equation:

(11) $${\left\|H\hat{\beta }-T\right\|}_{F}^{2}=\underset{\beta }{\min}{\left\|H\beta -T\right\|}_{F}^{2},$$

where ${\left\|.\right\|}_{F}$ is the Frobenius norm.

The high dimensionality and sparse characteristics of palmprint features may lead to the overfitting problem. In other words, the estimated weight, $\hat{\beta }$, may have a low bias and large variance leading to satisfactory performance on the training set, but deficient performance on the testing set. To overcome this problem, regularization is usually used. Regularization is normally an effective method for the reduction of such a problem [46]. This is done by sacrificing a little bias to shrink the variance of the predicted values. Consequently, the overall prediction accuracy is improved significantly.

There are some regularization methods that are used for the linear system such as the ridge regression, elastic net, lasso, nonconvex regularizer Lp (1/2<p<0), and minimax concave term. Nevertheless, these methods, except ridge regression, need an iterative estimated algorithm. When we take the advantage of the linear system in ELM, the Frobenius norm is used as a regularizer and Equation (11) can be rewritten as:

(12) $${\left\|H\hat{\beta }-T\right\|}_{F}^{2}=\underset{\beta }{\min}\left({\left\|H\beta -T\right\|}_{F}^{2}+\lambda {\left\|\beta \right\|}_{F}^{2}\right)$$

where λ is a regularizer parameter, there is a trade-off between the approximated error and the regularized degree, and $\hat{\beta }$ can be obtained by Theorem 1.

**Theorem** **1.** *The value of regularization in Equation (12) can be minimized to be the optimum solution when the regularizer parameter, λ, has a positive constant value in the following equation:*

(13) $$\hat{\beta }={\left(H^{T}H+\lambda I\right)}^{-1}H^{T}T.$$

**Proof.**  Suppose that the objective function of Equation (12) is given by: $l\left(\beta \right)\;={\left\|H\beta -T\right\|}_{F}^{2}+\lambda {\left\|\beta \right\|}_{F}^{2}.$ We take the derivative of l(β) and make it equal to zero as: $\frac{dl\left(\beta \right)}{d\beta }=0,$

Then, we get:

(14) $$\left(H^{T}H+\lambda I\right)\beta =H^{T}T$$

Since the matrix, $\left(H^{T}H+\lambda I\right)$ can be inverted where λ>0.

Then,

(15) $$\beta ={\left(H^{T}H+\lambda I\right)}^{-1}H^{T}T$$

The second derivative of l(β) with respect to β computed by:

(16) $$\frac{d^{2}l\left(\beta \right)}{d\beta \beta^{T}}=2\left(H^{T}H+\lambda I\right),$$

Consequently, Equation (13) is the optimum solution of Equation (12). Further discussion of the ELM optimization with different solutions is also introduced in [46]. ☐

## 4. Proposed Palmprint Recognition Approach

**Figure 2:**
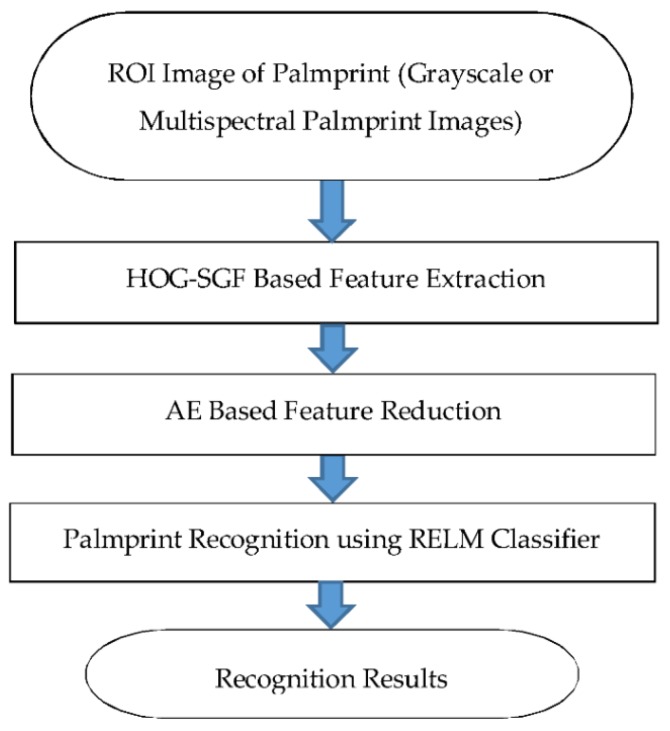
Flowchart of the proposed palmprint recognition approach.

The novelty and originality of the proposed approach comes from the new architecture used to recognize individuals using palmprint images. The approach starts after segmenting the ROI image from the input palmprint image using David Zhang’s method [5]. Therefore, the steps of the proposed approach include feature extraction, dimensionality reduction, and classification. Figure 2 exhibits the flowchart of the proposed approach with its main steps. In the feature extraction step, the hybrid HOG-SGF method was used to extract palm features, generating a vector with 1812 features.

After that, the AE method is utilized for reducing the dimensionality of the extracted feature vector into a small compressed vector containing only the most prominent features. Correct identification or recognition of individuals can be achieved when the compressed feature vector of the test palmprint image is closely similar to the compressed palmprint feature vector of the same user in the training dataset. Misidentification of a user may occur when the compressed feature vector of a test palmprint image is not similar to any compressed feature vector of palmprint images in the training dataset. The recognition step of the proposed approach was performed using RELM classifier. The motivation of using the RELM classifier is that the RELM is more robust to noise, changes, and variations than the traditional ELM, as well as fast enough for training and testing.

### 4.1. HOG-SGF Based Feature Extraction

The HOG feature method was initially introduced by Dalal and Triggs [47] to enhance the accuracy of human detection. The HOG descriptor was adopted in our study because it has some advantages over other descriptors. For example, the performance evaluation of HOG and Gabor features for vision-based detection [48] proved that HOG features provide more accuracy with less processing time compared to Gabor features. Furthermore, the HOG descriptor operates on local cells which make it more invariant to photometric and geometric transformation. The basic idea behind HOG features is that the local appearance and shape of the object in an image can be described by the intensity of gradients and their normalized histograms. The first step of the HOG method is to compute the gradient of each pixel in the input image to produce the gradient image. Then, the gradient image is divided into grids with 8 × 8 pixels, called cells. After that, a sliding window of size 16 × 16 pixels is passed through the cells to form overlapping blocks. Next, each pixel in a block is voted for based on its gradient orientation into a histogram. Finally, the histograms of each block are locally normalized and concatenated to form the HOG feature vector. Even though the normalized HOG feature vector is more robust to changes in illumination and shadowing of the input image, it is not rotational invariant. To solve this issue, we propose a hybrid method, named HOG-SGF. The HOG-SGF feature extraction method combines the HOG handcrafted features with the standard deviation and mean values of the SGF responses as additional features, summarizing the palmprint features at different rotation angles. Although deep learning based approaches are recently becoming very effective for feature extraction in many applications. We use the handcrafted features which are still suitable, which are very important for the palmprint recognition system for two reasons:

(1) The size of training dataset samples

Deep learning approaches usually require a lot of training samples for each class, especially in the large number of classes, to train the system model effectively. In such a system, there is a small number of samples for each user compared with the large number of users. This issue can be solved using a transfer learning concept. The applicability of the transfer learning concept for palmprint recognition can be investigated as a future direction for researchers to show the performance of deep and transfer learning techniques for recognizing individuals using their palmprint images.

(2) The cost of training and retraining the model

In the palmprint recognition system, sometimes we need to add or delete a user from the system model which requires it to be retrained. In this case, the retraining procedure of the deep learning model is computationally expensive.

In the following subsections, we explain the main steps of the hybrid HOG-SGF feature extraction method in more details.

#### 4.1.1. Dividing the Input Image into Cells and Blocks

**Figure 3:**
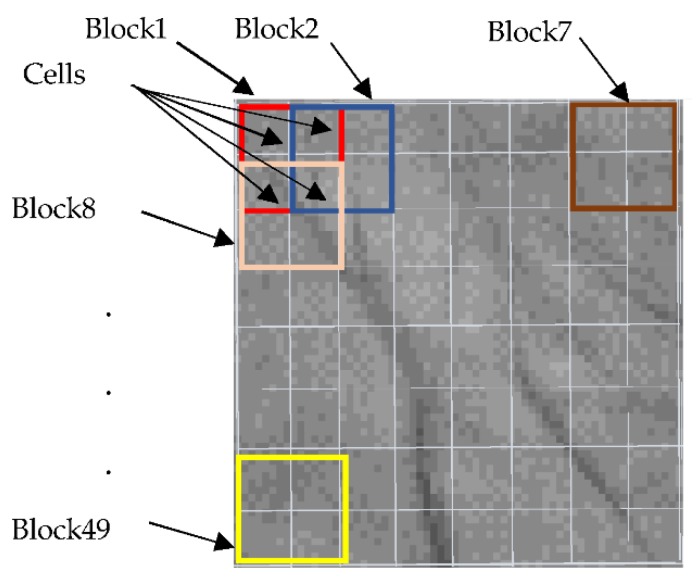
Dividing the palmprint image into cells and overlapping blocks.

In this step, the input palmprint image is scaled to 64 × 64 pixels and converted to a grayscale image. The resulting grayscale image is divided into cells of size 8 × 8 pixels. These cells are divided into blocks where each block consists of four neighboring cells as shown in Figure 3. Therefore, a total of 49 overlapping blocks can be generated to extract the features in the subsequent steps.

#### 4.1.2. Computing the Gradients’ Orientation

The gradient of each pixel is calculated using the following equations:

(17) dx=I(x+1,y)−I(x,y)

(18) dy=I(x,y+1)−I(x,y),

where dx and dy are the horizontal and vertical gradients, respectively. I(x,y), is the pixel value in the coordinate (x, y) of an image, I.

Then, the gradient orientation, θ is calculated by using Equation (19):

(19) $$\theta =tan^{-1}\left(\frac{dx}{dy}\right)$$

#### 4.1.3. Constructing the Histograms of the Gradients’ Orientation

In this step, the histograms of gradient orientations are constructed for each block based on different numbers of orientation bins. It is important to know that the higher number of bins will give more orientation details of the palm image. However, this will produce a large set of features and slow down the time of processing. For our method, the number of bins is nine to give a balance between the extracted orientation details and the size of extracted features.

#### 4.1.4. Block Normalization and Concatenation

Once the histograms of each block are produced, they are locally normalized to mitigate the variance of gradient strengths of cells within that block. When $B_{h}^{k}$ is the feature vector of the combined histograms (h) of the block region (k), the normalized blocks are computed by Equation (20) and concatenated by Equation (21) to form the HOG feature vector. We add a very small value, ε, to the denominator of Equation (20) to ensure that it is not equal to zero. Intuitively, every block of the 49 blocks has four cells, producing four histograms of nine bins, therefore it generates a block feature vector ($B^{k}$) with 36 values. The total size of $F_{\mathrm{HOG}}$ has a value of 49 × 36 = 1764.

(20) $$B^{k}=\frac{B_{h=1\ldots 36}^{k}}{\sqrt{\sum_{h=1}^{36}{\left|B_{h=1\ldots 36}^{k}\right|}^{2}}+\varepsilon }$$

(21) $$F_{\mathrm{HOG}}=F_{\mathrm{HOG}}\cup F^{k},$$

where k=1…49.

#### 4.1.5. Creating the Kernels of Steerable Gaussian Filter (SGF)

In this step, the kernels of SGF which are orientation-selective convolution kernels can be created by a linear combination of rotated forms of itself [49]. Basis filters are the partial derivatives of a 2D Gaussian with respect to x and y. At any given angle (θ), the creating process of the oriented filter is known as the *steering* process. The number of angles used for creating the kernels with respect to the image rows are 24 angles ($\theta_{i\in \left\{1,2,3,\ldots ,24\right\}}$ = {$0^{{}^{\circ}},\;15^{{}^{\circ}},\;30^{{}^{\circ}},\ldots ,\;345^{{}^{\circ}}\}$).

#### 4.1.6. Extracting Mean and Standard Deviation Features from the Filter Responses of an Image

**Figure 4:**
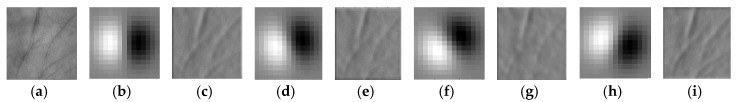
Oriented filter responses: (**a**) is a ROI of input palmprint image, (**b**,**c**) are filter kernels and filter responses when $\theta =0^{{}^{\circ}}$, (**d**,**e**) are filter kernels and filter responses when $\theta =15^{{}^{\circ}}$, (**f**,**g**) are filter kernels and filter responses when $\theta =30^{{}^{\circ}}$… (**h**,**i**) are filter kernels and filter responses when $\theta =345^{{}^{\circ}}$.

After creating the kernels of SGF, the oriented filter responses ($FR_{i}$) are computed by convolving the palmprint image with the filter kernels as shown in Figure 4 and using Equation (22):

(22) $$FR_{i}=\;cos\left(\theta_{i}\right)*Ix+sin\left(\theta_{i}\right)*Iy,$$

where i=1,2,3,…,24, Ix and Iy are the image gradients with respect to image rows (x) and columns (y).

The mean and standard deviation of each filter response were computed and used as features to summarize the edge responses of the palm images at different angles, generating a SGF feature vector ($F_{\mathrm{SGF}}$) of size 48 feature value as follows:

(23) $$\mu_{i}=\;mean\left(FR_{i}\right)$$

(24) $$\sigma_{i}=\;std\left(FR_{i}\right)$$

(25) $$F_{\mathrm{SGF}}=F_{\mathrm{SGF}}\cup \mu_{i}\cup \sigma_{i},$$

where i=1,2,3,…,24.

In the end of this step, the SGF feature vector ($F_{\mathrm{SGF}}$) was combined with the HOG feature vector ($F_{\mathrm{HOG}}$) to form a feature vector ($F_{HOG-SGF}$) of size 1812 feature value as follows:

(26) $$F_{HOG-SGF}=F_{\mathrm{HOG}}\cup F_{\mathrm{SGF}}$$

#### 4.1.7. Feature Vector Normalization

Here, the HOG-SGF feature vector ($F_{\mathrm{HOG}-\mathrm{SGF}}$) is normalized using Euclidean norm as in Equation (27), obtaining the normalized feature vector ($NF_{\mathrm{HOG}-\mathrm{SGF}}$). By this, we reduced the global effect of feature variations in the entire feature vector.

(27) $$NF_{\mathrm{HOG}-\mathrm{SGF}}=\frac{F_{i=1\ldots 1812}}{\sqrt{\sum_{i=1}^{1812}{\left|F_{i}\right|}^{2}}}$$

We chose the Euclidean norm because it is the natural norm associated with the dot-product that measures the similarity between objects.

### 4.2. AE Based Feature Reduction

A nonlinear AE is practically used to reduce the dimensionality of the HOG-SGF features. It is applied for producing new features from the extracted features of training and test data sets, separately. There are several possible parameters for AE that should be initialized and tuned during this step. The significant parameters include: the number of hidden nodes, the activation function (such as a sigmoid, tanh, softmax and ReLU activation functions), the regularization parameters or weight decay terms of hidden unit weights, the learning rate, and the number of epochs to be iterated. Now, suppose the inputs, $X\epsilon {ℛ}^{1812}$ in Equation (1), are the values of the extracted HOG-SGF features, and the outputs, $\hat{X}\epsilon {ℛ}^{1812}$, are reconstructed values by Equation (2). Since there are only *K* hidden units, the AE is forced to learn a compressed representation, $Z\epsilon {ℛ}^{K}$ (new features), to reconstruct the 1812-features of input *X*. These new features will be fed to the RELM for classification.

### 4.3. Palmprint Recognition Using RELM Classifier

The RELM classifier for classifying users’ palmprints is based on the training samples in the feature space created from ae. It is one of the fastest and most accurate algorithms in the field of machine learning. Generally, RELM focuses on function approximation to classify specific instances. According to the classification problem of the users’ palmprint, we need a user id discrimination function. Here, we encode the user id as a target vector, and in order to represent this encoding uniformly, we defined the target vector corresponding to users ids $\left(id_{j}\right)$ as:

(28) $$t_{j}={\left(b_{1},\ldots ,b_{i},\ldots ,\;b_{m}\right)}^{T}$$

where *m* is the number of users in the training set, and $b_{i}$ is equal to 1 or −1, depending on whether the related user ID belongs to the corresponding IDs or not.

For example, suppose that there are three users (m = 3), the users’ IDs, $id_{j\epsilon \left(1,\;2,\;3\right)\;}=\left(1,2,3\right)$, and the related target vectors: $t_{1}={\left(1,-1,-1\right)}^{T}$ belong to the first user, $t_{2}={\left(-1,\;1,-1\right)}^{T}$ belongs to the second user, and $t_{3}={\left(-1,\;-1,\;1\right)}^{T}$ belongs to the third user. For the test set, the output target matrix can be evaluated as:

(29) $$Y=\hat{H}\hat{\beta },$$

where $\hat{H}$ is the hidden layer output matrix of testing data. According to the users IDs (multi-label), we define the user ID discrimination function as:

(30) $$id_{j}=\underset{i}{arg}max\left(Y_{j}\right),$$

Algorithm 1 summarizes the main phases and steps of recognition process in the proposed approach.

**Algorithm 1.** Palmprint Recognition Using RELM ClassifierInput: the reduced features of training and testing set and setting parameters Output: the labels of testing set Learning stage: 1: Initializing the weights and biases of RELM randomly 2: Computing the matrix, *H* of the hidden layout using Equation (8) 3: Computing the matrix, *T* of the hidden layer using Equation (9) 4: Computing the output weights, $\hat{\beta }$ using Equation (13) Classification stage: 5: Computing the matrix, $\hat{H}$ of the hidden layout using Equation (8) 6: Computing the output weights, *Y* using Equation (29) 7: Classifying the testing user ID using Equation (30) depending on whether this ID belongs to the user ID in the training set.

## 5. Experiment and Discussion

A comprehensive set of experiments is conducted on three databases of palmprint images. At first, experiments on contact multispectral palmprint images were conducted using the MS-PolyU [5] database. For further evaluation, and assessment of the proposed approach, experiments on grayscale palmprint images were also conducted using CASIA [50] and Tongji [30] databases of contactless palmprint images. The description of the palmprint databases and the explanation of the experiments with results and comparisons are given in the next subsections.

### 5.1. Description of Palmprint Databases

**Figure 5:**
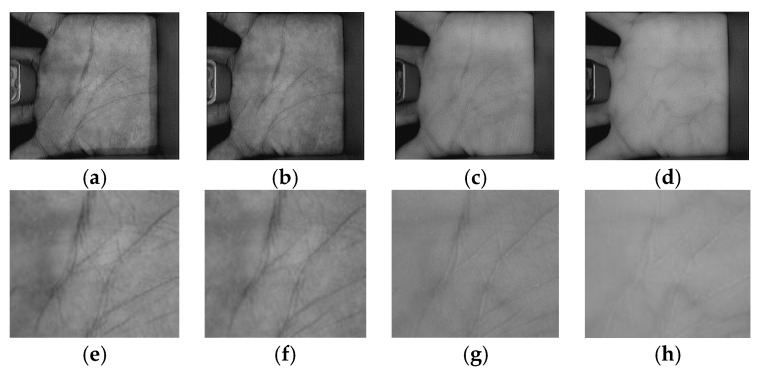
Spectral palmprint images of the same palm taken from the MS-PloyU database: (**a**–**d**) are palmprint images of blue, green, red, and near-infrared (NIR) bands, and (**e**–**h**) are ROI images corresponding to (**a**–**d**), respectively.

Three benchmark databases of palmprint images were used to evaluate the results of this study. The first database was collected by Hong Kong Polytechnic University (PolyU) [5]. The multispectral PolyU (MS-PolyU) database contains 24,000 contact palms images of 250 different people, each with two different palms, captured under four different illuminations namely: red, green, blue, and NIR spectra types. These images were collected from 195 males and 55 females in two different sessions. In each session, 6 images were provided for each spectrum. Approximately nine days was the average interval time between the first and the second session. Hence, there were 250 (volunteers) × 2 (different palms) × 4 (different spectra) × 6 (images) × 2 (sessions) = 24,000 images in this database. Figure 5 shows sample spectral palmprint images of the same palm with their ROIs taken from the MS-PolyU database in red, green, blue, and NIR spectral bands.

**Figure 6:**
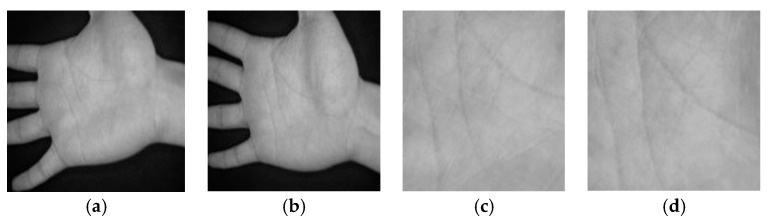
Sample grayscale images of the same palm taken from the CASIA database: (**a**,**b**) are two typical contactless palmprint images and (**c**,**d**) are two ROI images corresponding to (**a**,**b**), respectively.

The second database is provided by the Chinese Academy of Sciences’ Institute of Automation (CASIA) [49]. The CASIA database contains about 5502 palmprint images collected from 312 persons. For each person, 8 palmprint images were collected from the left and right palms with varying positions and postures. So, the palmprint images of this database have significant variations in positions, postures, and scales. Two typical grayscale palmprint images of the same palm with their ROI images taken from CASIA database are shown in Figure 6.

**Figure 7:**
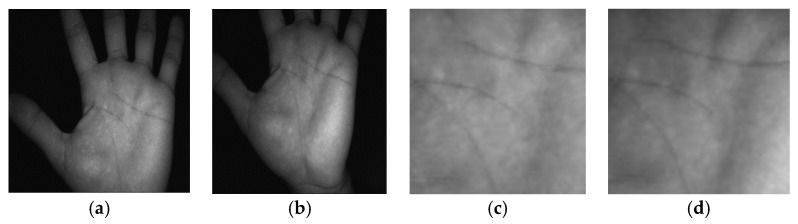
Sample grayscale images of the same palm taken from the Tongji database: (**a**,**b**) are two typical contactless palmprint images and (**c**,**d**) are two ROI images corresponding to (**a**,**b**), respectively.

A new large-scale database of contactless palmprint images, named the Tongji database, is also used in our experiments. The images of this database are captured from 300 volunteers (192 males and 108 females) of Tongji University [30]. The ages of the 235 volunteers were between 20 and 30 years, and the others were between 30 and 50 years. The samples were collected in two different sessions. The average time was around 61 days between the first and the second sessions. The minimum time interval was 21 days and the maximum time interval was 106 days. In each session, 10 images were captured from each palm of each subject. Hence, there were 40 images from 2 palms resulting in 6000 images for each session. Therefore, there were a total of 12,000 images collected from 600 different palms in the two sessions. Each palmprint image of this database has a size of 600 × 800 pixels. Figure 7 shows two typical palmprint grayscale images of the same palm taken from Tongji database.

### 5.2. Parameter Settings

There is a set of parameters for the experiments which need to be initialized. Table 1 states all the parameters of our experiments. For instance, the number of AE’s hidden nodes, $K_{AE}$ is fixed to 800, since it is enough to represent the most important extracted features of the palmprint. Specifically, we initialized the AE’s hidden nodes parameter, $K_{\mathrm{AE}}$ with four different values (200, 500, 600 and 800) in the procedure 1 to show their effect on the recognition rates. Another substantial parameter is the number of RELM’s hidden nodes, $K_{RELM}$, which is also chosen using a grid search technique. The grid search is performed on 18 different numbers of hidden nodes, between 800 and 1820, increased by 60 nodes every time. A nonlinear sigmoid activation function was also used in our experiments.

### 5.3. Experiment on Multispectral Palmprints

Experiments on multispectral palmprint images were performed by four different procedures to evaluate the efficiency and robustness of the proposed approach. The first three procedures were utilized to examine the accuracy of the proposed approach on each spectral band under different number of training and testing samples. The fourth procedure was used to show the advantage of feature combination of multiple bands for improving the accuracy of palmprint recognition system.

#### 5.3.1. Procedure 1

**Figure 8:**
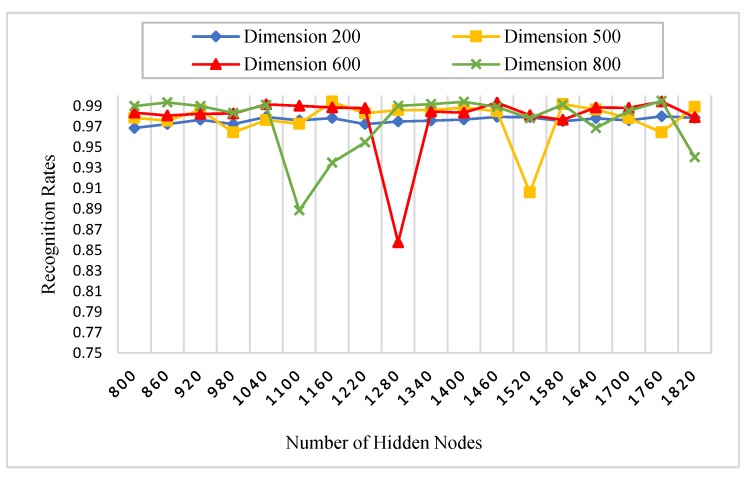
The effect of feature dimensions and numbers of RELM’s hidden nodes on recognition rates of the blue spectral band.

**Figure 9:**
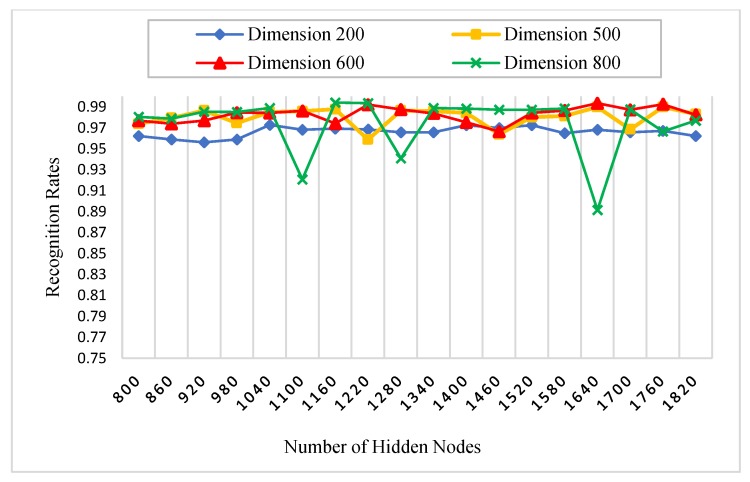
The effect of feature dimensions and numbers of RELM’s hidden nodes on recognition rates of the green spectral band.

**Figure 10:**
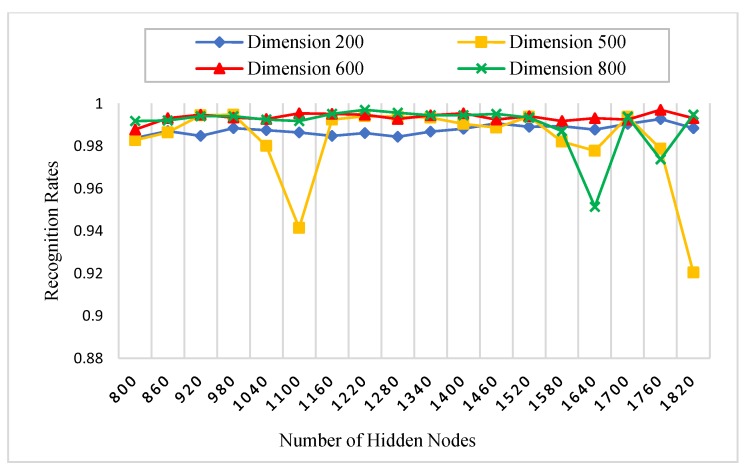
The effect of feature dimensions and numbers of RELM’s hidden nodes on recognition rates of the red spectral band.

**Figure 11:**
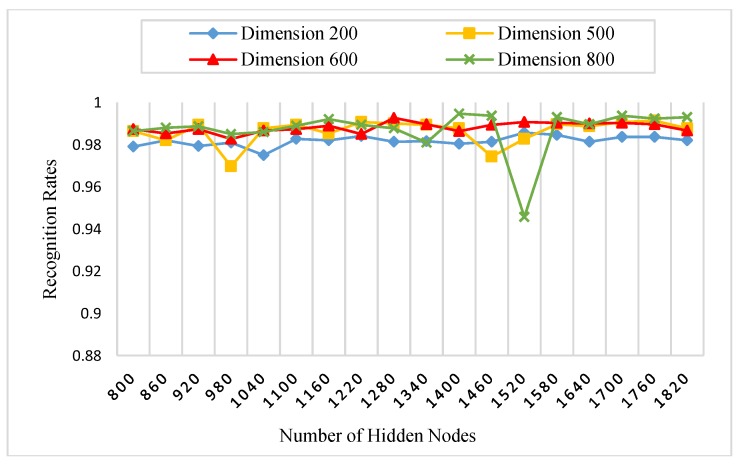
The effect of feature dimensions and numbers of RELM’s hidden nodes on recognition rates of the NIR spectral band.

In this procedure, six images of each palm from the first session were selected as a training dataset, whereas, all images of the second session are used as a testing dataset. The number of subjects was 250, and for each subject, two images were captured. Therefore, there were 3000 (500 × 6) training images and 3000 (500 × 6) testing images for each spectral band (red, green, blue, and NIR). Initially, we performed this procedure with different numbers of AE’s and RELM’s hidden nodes to study the effect of feature dimensions (number of AE’s hidden nodes) and the number of RELM’s hidden nodes on palmprint recognition rates. As stated in Table 1, the scaled conjugate gradient (SCG) function was used in the training algorithm of AE because it has the ability to learn the feature representation of palmprint images effectively, achieving high recognition rates. Figure 8, Figure 9, Figure 10 and Figure 11 show the recognition rates of all spectral bands using different feature dimensions and different RELM’s hidden nodes. In these Figures, we can notice some drops in the accuracy achieved by some feature dimensions at different RELM’s hidden nodes. This is due to the nonmonotonic nature of the feature in these dimensions when the RELM classifier transfers into a high dimensional domain according to the number of hidden nodes.

From Figure 8, Figure 9, Figure 10 and Figure 11, we see that the highest recognition rates were achieved by feature dimensions of 800 at different numbers of RELM’s hidden nodes. Furthermore, we see that the number of drops in accuracy regarding the feature dimension of 800 was very small compared to other dimensions for all spectral bands. Hence, this feature dimension is selected as a fixed size of feature reduction in all experimental procedures of this study.

**Figure 12:**
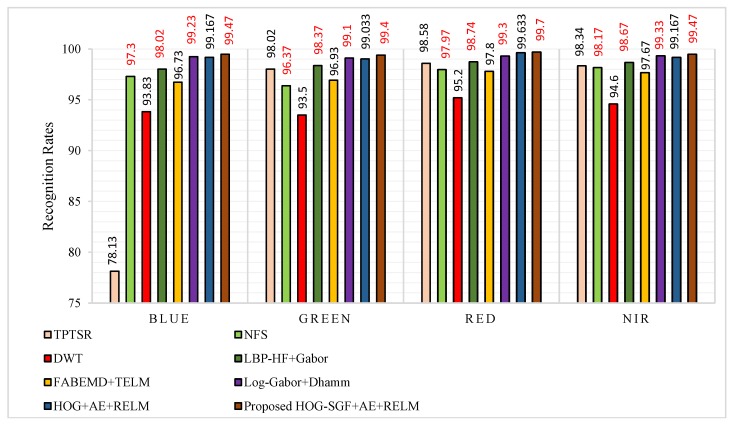
Recognition rates of procedure 1 for the proposed approach compared to TPTSR [13], NFS [14], DWT [16], FABEMD+TELM [23], LBP-HF+Gabor [24], Log-Gabor+DHamm [29] and HOG+AE+RELM approaches.

In the results of Table 2 and Figure 12, we seen that the proposed approach yields the highest recognition rates of 99.47% for the blue band, 99.40% for the green band, 99.70% for the red band, and 99.47% for the NIR band. These results are highlighted in a boldface font in Table 2. Furthermore, in the case of blue spectral band, there are improvements of 21.34% compared to TPTSR, 2.17% compared to NFS, 5.64% compared to DWT, and 1.45% compared to LBP-HF + Gabor. Additionally, the approach achieves improvements of 2.74% and 0.24% against the recent methods, FABEMD + TELM and Log-Gabor + D_Hamm_ methods, respectively. Moreover, we see the approach using the hybrid HOG-SGF feature extraction method achieves an improvement of 0.303% compared to the approach when using the HOG feature extraction method.

In the case of other bands, the results also show that the proposed approach obtains competitive results compared to the state-of-the-art approaches. Even though this procedure uses one session for training and another different session for testing which may be affected by different conditions, such as changes in illumination, shadowing, and orientation, the approach achieves interesting results. Clearly, this proves the robustness of the hybrid HOG-SGF method to changes in illumination and orientation problems, the ability of AE to deal with the nonlinearity of features, and the effectiveness of RELM to solve the overfitting problem, which gives more power to the proposed approach.

To verify the performance of the proposed approach, we also use Equal Error Rates (EERs) [5] as another evaluation metric. EERs are computed using the average of False Rejection Rates (FRRs) and False Acceptance Rates (FARs) at different threshold values ranging from 0 to 1. Typically, FARs can be mathematically calculated as the ratio of false acceptances divided by the number of user attempts. False acceptance is the case when the biometric system incorrectly accepts the user who is not enrolled as a legal user. In contrast, the FRRs can be measured as the ratio of false rejections divided by the number of user attempts. False rejection means that the system incorrectly rejects the user who is actually enrolled as a legal user. Table 3 shows the EERs of the proposed method compared to the orientation-based methods of state-of-the-art approaches: Palm code [5], BDOC–BHOG [6], Fusion code [26], Ordinal code [27], Competitive code [28], RLOC [31], BOCV [32], EBOCV [33], HOC [34], DOC [35], and Block-wise Gaussian Derivative Phase Pattern Histogram (BGDPPH) [51]. As seen in Table 3, the EER of the proposed HOG-SGF method is about 0.0040% for the NIR band, 0.0025% for the red band, 0.0113% for the green band, and 0.0073% for the blue band. It achieves the lowest EERs on all spectral bands against the other orientation-based methods as highlighted in boldface font in Table 3.

Furthermore, it can be noticed that the EERs of the proposed method for both red and NIR spectral bands are smaller than the EERs of green and blue bands. The main potential cause of this may be because the coarse features of green and blue bands are very sharp and need a level of smoothness.

#### 5.3.2. Procedure 2

This procedure is used to validate the robustness of this work in the case of a small number in training samples. Hence, the first three images of each different palm from the first session are used as a training dataset of 1500 (500 × 3) images, and the six images of each palm from the second session are used as a testing dataset of 3000 (500 × 6) images for each spectral band, separately. We compared the results of the approach with the approaches that used the same number of training and testing samples, such as NFS [14], RBF [15], and LBP-HF + Gabor [24]. Obviously, the experimental results in Table 4 demonstrate the strength of the proposed approach in terms of effectiveness and robustness over other reported approaches. We obtained attractive recognition rates of 98.767–99.600%, which are highlighted in a boldface font in Table 4. With regard to the recognition rates of blue, green, red, and NIR bands, it can be observed that the proposed approach yields improvements of 1.067%, 1.593%, 1.36%, and 0.63%, respectively, when compared to the LBP-HF + Gabor approach which has the highest recognition rates against other approaches of the state-of-the-art approaches. Similarly, the proposed approach based on HOG-SGF method achieves improvements of 0.467%, 1.6%, 0.4%, and 0.9% according to the blue, green, red, and NIR bands when compared to the proposed approach based on traditional HOG.

**Figure 13:**
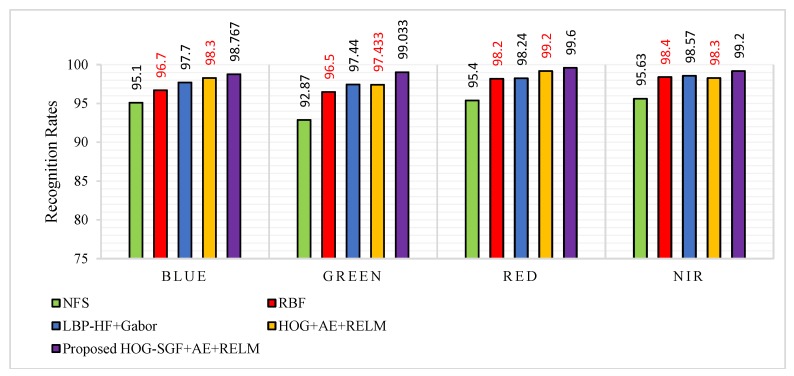
Recognition rates of procedure 2 for the proposed approach compared to NFS [14], RBF [15], LBP-HF+Gabor [24] and HOG+AE+RELM approaches.

The results in Table 4 provide additional proof of the performance of the method under a small number of training samples. These attractive recognition rates and satisfactory performances are due to the robustness of the HOG-SGF method with AE and RELM used to solve the palmprint identification problem under different environmental conditions with a small number of training samples. Figure 13 summaries and visualizes the recognition rates of procedure 2 against the best of recent works.

#### 5.3.3. Procedure 3

**Figure 14:**
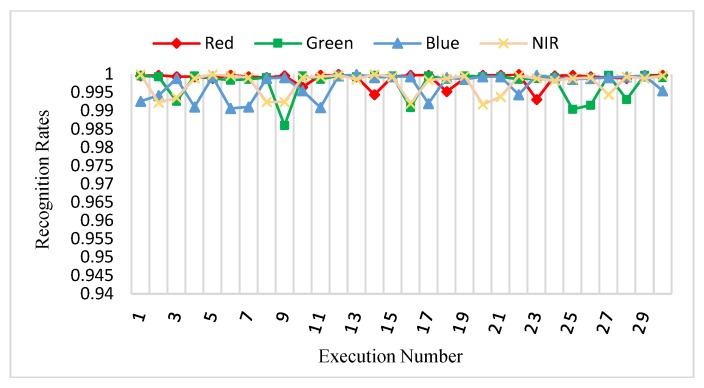
Recognition rates of the thirty runs of the procedure 3.

Procedure 3 of our experiment follows a previously described method [22]. Here, from the two sessions, three samples of each different palm were randomly chosen as a training dataset of 1500 (500 × 3) images and the other remaining nine samples of each different palm are considered as a testing dataset of 4500 (500 × 9) images for each spectral band. This procedure was repeatedly performed thirty times, and the final results are shown as the average recognition rates of these thirty times. Figure 14 shows the recognition rates of this procedure.

**Figure 15:**
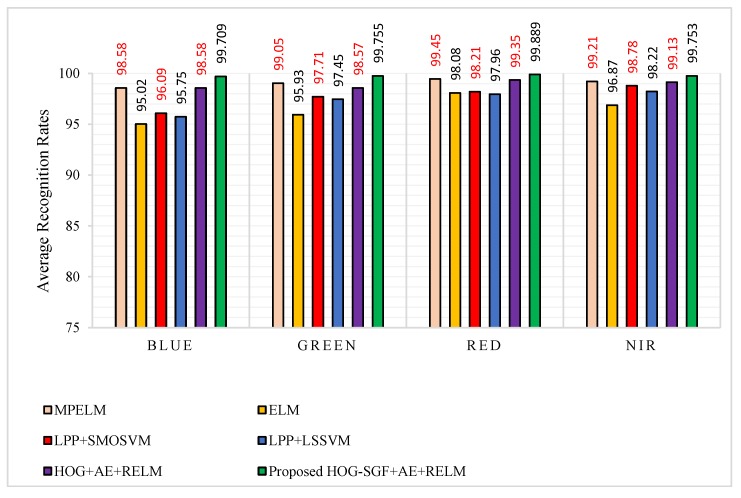
Average recognition rates of procedure 3 for the proposed approach compared to MPELM [22], ELM [22], LPP+SMOSVM [22], LPP+LSSVM [22] and HOG+AE+RELM approaches.

As highlighted in boldface font in Table 5 and shown in Figure 15, the proposed approach achieves a high average recognition rates of 99.709%, 99.755%, 99.889%, and 99.753%, regarding to blue, green, red, and NIR spectral bands, respectively. Additionally, it can be observed that the proposed approach yields improvements of 1.129%, 0.705%, 0.439%, and 0.543% for blue, green, red, and NIR spectral bands, compared to the MPELM approach. Furthermore, we see that the accuracy of the proposed approach using the HOG-SGF method is better than that using the HOG descriptor, especially for blue and green spectral bands which have more variations in illumination and shadowing. For analyzing accuracy based on spectral bands, the results indicate that the red spectral band outperforms all other spectral bands, whereas the green and NIR spectral bands perform better than the blue spectral band.

#### 5.3.4. Procedure 4

In this paper, we aim to show the advantage of combining multiple band features for improving the accuracy of a palmprint recognition system. To achieve this aim, we combined the HOG-SGF features of the NIR band with the features of the other bands separately. In other words, the HOG-SGF features of blue with NIR, green with NIR, and red with NIR bands were used for the recognition of palmprint images. The advantage of such combinations was worth investigation in this study. With respect to the number of training and testing samples used for evaluation, we followed the same number of training and testing samples in procedures 1 and 3. The experimental results of this procedure were compared to the latest works which were conducted on the MS-PolyU database. The results of these comparisons are presented in Table 6 and Table 7.

From Table 6 and Table 7 we see that the proposed approach attains higher recognition rates than the other methods for all multiple bands. These highest recognition rates are highlighted in boldface font in Table 6 and Table 7. In more detail, we notice that the combination of blue + NIR and green + NIR is more useful to achieve a high improvement than the combination of Red + NIR. The reason for this improvement is due to the fine features of NIR which smooths the coarse features of blue giving more distinctive information for differentiating between different palmprint images effectively.

### 5.4. Experiment on Grayscale Palmprints

We conducted additional experiments on the contactless palmprint images of CASIA and Tongji databases for a greater evaluation of the proposed approach. For the CASIA database, the first 103 subjects were selected for generating the training and testing datasets. Subjects which had numbers 63, 70, and 75 were discarded because they have no images or fewer images than the other subjects. Therefore, the experiment was conducted on 100 subjects, each having eight images from the left palm and eight images from the right palm which resulted in 1600 palmprint images. From these images, two and six images for each palm were randomly selected as a training dataset and the remaining palmprint images were used as a testing dataset. Table 8 compares and shows the results of the proposed method with six methods of the state-of-the-art approaches, which were the Competitive Code [28], Orthogonal line ordinal features with Scale invariant feature transform (OLOF + SIFT) [38], Sparse subspace clustering (SSC) [52], Gaussian fields and harmonic functions (GFHF) [53], Low-rank representation integrated with principal line distance (LRRIPLD) [2], and the traditional HOG method.

From Table 8, we see that the proposed approach using the HOG-SGF method attains higher accuracy results than the other methods for both two and six training samples of the CASIA database. The highest accuracy results achieved by the proposed approach are highlighted in boldface font in Table 8. Even though the improvement of the proposed approach on the contact of the MS-PolyU database, which has small variations in illuminations and positions, is very limited, the proposed approach achieves a significant improvement on the contactless CASIA database which has a high variation in positions, postures, and scales. This confirms the robustness of the proposed method and shows its improvement in the presence of variations in illuminations and rotations.

In the Tongji database, the palmprint images are collected from 300 subjects in two different sessions. For each session, 10 images were captured from each palm of each subject to result in 6000 images. Hence, there are in total 12,000 images for both sessions. Among these two sessions, the collected images of the first session were selected as a training dataset and the images of the second session were used as a testing dataset. Comparing with a recent work conducted on the Tongji database, Table 9 illustrates the results of accuracy and the time cost under the same experimental setup and machine.

The experimental results of the Tongji database show that the proposed approach using the HOG-SGF method achieves higher accuracy compared to the accuracy of HOG, and a competitive accuracy with a low time cost compared to the CR_CompCode. The value in boldface font in Table 9 represents this highest accuracy result which is obtained by our proposed approach. Finally, from all experiments, we can say that the proposed approach using the HOG-SGF method achieves the best results compared to the state-of-the-art approaches. On the other hand, the accuracy of the HOG-SGF method was always higher than the accuracy of HOG, which proves that the HOG-SGF method was able to extract the palmprint features in more orientations than the HOG method.

### 5.5. Computational Efficiency

The experiments were implemented using MATLAB R2015a on a laptop with Windows 10 (x64), Intel(R) Core(TM) i7-4510U CPU (2.0 GHz), and 8 GB RAM. The average execution time required to perform feature extraction, feature reduction, and classification was a way of measuring the computational efficiency of the proposed technique. Therefore, we computed the average execution time of feature extraction, feature reduction, and classification, as listed in Table 10, Table 11 and Table 12.

We noticed that the execution time of the feature extraction based on HOG and HOG-SGF was almost equal. The execution time of the feature reduction based on AE increased, with an increase in the number of hidden nodes, which represented the dimensionality of reduced features.

Moreover, we observed that the training time of the AE + RELM model slightly increased when increasing the number of RELM’s hidden nodes, whereas the testing time was very small, and did not increase dramatically when increasing the number of dimensions and RELM’s hidden nodes. In general, as shown in Table 10, Table 11 and Table 12, the approach was efficient and fast enough for a real-time palmprint recognition system.

For further analysis of the computational efficiency for the proposed technique, one image with a size of 64 × 64 pixels required 0.00955 s for feature extraction, 0.00961 s for pre-training, 0.00141 s for training, and 0.00875 s for testing, on a laptop with the same specifications mentioned in the beginning of this subsection.

In term of time complexity (Big-O notation), the overall average time of the proposed technique was a constant time, C, for processing 1 image and C×n for processing n images. Therefore, the computational time of the proposed technique is a linear time, O(n).

## 6. Conclusions and Future Work

A novel palmprint recognition approach is proposed based on AE and RELM with a hybrid feature extraction method, named HOG-SGF. The hybrid HOG-SGF method was applied to extract the features of palmprint, while AE was used to solve the problem of high dimensionality associated with the HOG-SGF features. The recognition rate of the proposed approach has been evaluated using three benchmark databases of palmprint images. These databases were MS-PolyU of contact multispectral palmprint images, CASIA, and Tongji of visible contactless palmprint images, taken by the natural light imaging system. The experiments were divided into two groups. The first group of experiments was conducted on the multispectral palmprint images through four different procedures. The second group of experiments was conducted on the grayscale palmprint images of the contactless visible natural light imaging system.

From all experiments, the results proved that the accuracy of the proposed approach using the HOG-SGF method is better than when using the HOG descriptor. On the other hand, the results showed that the proposed approach achieved higher recognition rates when compared to the recent works of the state-of-the-art approaches. Moreover, it has been observed that the red spectral band outperforms all other bands effectively in the first three procedures of the experiments, whereas the features combination of green + NIR and blue + NIR bands achieved better results in the last experimental procedure. However, the result of our approach using red + NIR also achieved good recognition rates compared to the latest works in the literature review. Moreover, the experimental results of CASIA and Tongji databases of the contactless grayscale palmprint images confirmed and proved the performance of the proposed approach against the works which were conducted on these databases of palmprint images. Our next step is to extend the approach to handle spoofing problems by using multispectral palmprint image fusion methods and deep learning techniques. We will also apply the proposed HOG-SGF algorithm on some applications which have images of different colors and structures.

## Figures and Tables

**Table 1:** Parameters Settings.

Method	Parameters
HOG-SGF	Image Size = 64 × 64 = 4096 pixels. Block Size = 2 × 2 = 4 cells. Number of Bins (HOG orientations) = 9 bins. Cell Size = 8 × 8 = 16 pixels. Number of Blocks per Image = 7 × 7 = 49 blocks. Number of SGF rotated angles = 24.
AE	Number of Hidden Nodes, $K_{AE}\in \left\{200,\;500,\;600,\;800\right\}$. Encoder and Decoder Transfer Function is a Logistic Sigmoid Function. Maximum Epochs = 10. L2WeightRegularization = 0.004. Loss Function is a Mean Squared Error function. Training Algorithm is based on a Scaled Conjugate Gradient Function.
RELM	A Number of Hidden Nodes is, $K_{RELM}\in \left\{800,\;860,\;920,\ldots ,1820\right\}.$ Regularization parameter is: (λ) = exp(val), where val∈{−1,−0.9,−0.8,…,0.9, 1} An Activation function is a Nonlinear Sigmoid Function, $g\left(x\right)=\left(\frac{1}{1+e^{-x}}\right)$.

**Table 2:** Recognition rates of NIR, red, green, and blue spectral bands for the proposed approach compared to some approaches in the literature using procedure 1.

Approach [Ref.]	Recognition Rates (%)			
	Blue	Green	Red	NIR
TPTSR [13]	78.13	98.02	98.58	98.34
NFS [14]	97.30	96.37	97.97	98.17
DWT [16]	93.83	93.50	95.20	94.60
LBP-HF+Gabor [24]	98.02	98.37	98.74	98.67
FABEMD+TELM [23]	96.73	96.93	97.80	97.67
Log-Gabor+D_Hamm_ [29]	99.23	99.10	99.30	99.33
HOG+AE+RELM	99.167	99.033	99.633	99.167
Proposed HOG-SGF+AE+RELM	**99.47**	**99.40**	**99.70**	**99.47**

**Table 3:** EERs of the proposed HOG-SGF method compared to other orientation-based methods.

Methods [Ref.]	ERRs (%)			
	Blue	Green	Red	NIR
Competitive code [28]	0.0170	0.0168	0.0145	0.0137
Palm code [5]	0.0463	0.0507	0.0297	0.0332
Fusion code [26]	0.0212	0.0216	0.0179	0.0213
Ordinal code [27]	0.0202	0.0202	0.0161	0.0180
BDOC–BHOG [6]	0.0487	0.0418	0.0160	0.0278
RLOC [31]	0.0203	0.0249	0.0223	0.0208
BOCV [32]	0.0207	0.0232	0.0186	0.0284
EBOCV [33]	0.0225	0.0303	0.0313	0.0510
HOC [34]	0.0147	0.0144	0.0131	0.0139
DOC [35]	0.0146	0.0146	0.0119	0.0121
BGDPPH [51]	0.4100	0.4600	0.2900	0.4000
HOG-SGF	**0.0073**	**0.0113**	**0.0025**	**0.0040**

**Table 4:** Recognition rates of NIR, red, green, and blue spectral bands for the proposed approach compared to some approaches in the literature using procedure 2.

Approach [Ref.]	Recognition Rates (%)			
	Blue	Green	Red	NIR
NFS [14]	95.10	92.87	95.40	95.63
RBF [15]	96.70	96.50	98.20	98.40
LBP-HF+Gabor [24]	97.70	97.44	98.24	98.57
HOG+AE+RELM	98.300	97.433	99.200	98.300
Proposed HOG-SGF+AE+RELM	**98.767**	**99.033**	**99.600**	**99.200**

**Table 5:** Average recognition rates of NIR, red, green, and blue spectral bands for the proposed approach compared to some approaches in the literature using procedure 3.

Approach [Ref.]	Average Recognition Rates (%)			
	Blue	Green	Red	NIR
MPELM [22]	98.58	99.05	99.45	99.21
ELM [22]	95.02	95.93	98.08	96.87
LPP+SMOSVM [22]	96.09	97.71	98.21	98.78
LPP+LSSVM [22]	95.75	97.45	97.96	98.22
HOG+AE+RELM	98.58	98.57	99.35	99.13
Proposed HOG-SGF+AE+RELM	**99.709**	**99.755**	**99.889**	**99.753**

**Table 6:** Recognition rates of the proposed approach against the state-of-the-art approaches of the combinations of NIR band with other bands, based on selecting the first session images for training and the second session images for testing.

Approach [Ref.]	Recognition Rates (%)		
	Blue + NIR	Green + NIR	Red + NIR
FABEMD+TELM [23]	99.10	99.47	99.47
Log-Gabor+D_Hamm_ [29]	99.63	99.67	99.50
Log-Gabor+D_KL_ [29]	99.60	99.63	99.47
Proposed HOG-SGF+AE+RELM	**99.90**	**99.77**	**99.80**

**Table 7:** Recognition rates of the proposed approach against the state-of-the-art approaches on the combinations of NIR band with other bands, based on randomly selecting three images for training and nine images for testing repeated thirty times.

Approach [Ref.]	Recognition Rates (%)		
	Blue + NIR	Green + NIR	Red + NIR
MPELM [22]	99.17	99.51	99.56
ELM [22]	97.46	97.98	98.41
LPP+SMOSVM [22]	98.38	98.51	98.93
LPP+LSSVM [22]	98.62	99.05	99.21
Proposed HOG-SGF+AE+RELM	**99.99**	**99.90**	**99.95**

**Table 8:** Accuracy of the proposed approach using the HOG-SGF method compared to some methods from the CASIA database of palmprint images.

Approach [Ref.]	Classifier	Accuracy (%)	
		2 Samples of Training	6 Samples of Training
Competitive Code [28]	Hamming distance	77.12	90.55
OLOF+SIFT [38]	Euclidean distance	75.85	91.77
SSC [52]	Euclidean distance	40.70	86.60
GFHF [53]	Euclidean distance	80.61	89.52
LRRIPLD [2]	Principal line distance	86.75	95.05
HOG+AE	RELM	87.52	95.67
Proposed HOG-SGF+AE	RELM	**91.95**	**97.75**

**Table 9:** Accuracy and time cost of the proposed approach using HOG-SGF method compared to the recent work on Tongji database of palmprint images.

Approach	Classifier	Time Cost (s)		
		Feature Extraction of One Image	Recognition of One Image	Accuracy (%)
CR_CompCode [30]	Euclidean distance	0.0150	0.0247	98.78
HOG+AE	RELM	0.00274	0.0088	97.2
Proposed HOG-SGF+AE	RELM	0.00955	0.0088	**98.85**

**Table 10:** Average execution time of feature extraction.

Method	Avg. Time (s)
HOG based feature extraction	0.00274
HOG-SGF based feature extraction	0.00955

**Table 11:** Average execution time of feature reduction using two different numbers of hidden nodes.

Method	AE’s Hidden Nodes	Avg. Time (s)
Pre-training of AE Model on 3000 images	200	6.2725
Pre-training of AE Model on 3000 images	800	28.8237

**Table 12:** Average execution time of training and testing using two different numbers of feature dimensions and hidden nodes.

Method	Feature Dimensions	RELM’s Hidden Nodes	Avg. Time (s)
Training of AE+RELM Model on 3000 images	200	800	1.18804
Training of AE+RELM Model on 3000 images	800	800	1.23685
Training of AE+RELM Model on 3000 images	200	1820	3.42992
Training of AE+RELM Model on 3000 images	800	1820	4.24177
Testing of AE+RELM Model on a one test image	200	800	0.00610
Testing of AE+RELM Model on a one test image	800	800	0.00840
Testing of AE+RELM Model on a one test image	200	1820	0.00656
Testing of AE+RELM Model on a one test image	800	1820	0.00875
